# Clinical Presentation, Causes, Treatment, and Outcome of Lip Avulsion Injuries in Dogs and Cats: 24 Cases (2001–2017)

**DOI:** 10.3389/fvets.2018.00144

**Published:** 2018-07-06

**Authors:** Kelly M. Saverino, Alexander M. Reiter

**Affiliations:** Department of Clinical Studies, Dentistry and Oral Surgery Service, Matthew J Ryan Veterinary Teaching Hospital, University of Pennsylvania, Philadelphia, PA, United States

**Keywords:** lip, avulsion, degloving, canine, dog, feline, cat, trauma

## Abstract

Lip avulsions are a common result of orofacial trauma in dogs and cats. Vehicular trauma and bite wounds are common causes. Surgical therapy is highly successful with early decontamination and tension-free closure. This retrospective case series assessed the signalment, causes, lesion location, treatment and outcome of lip avulsion injuries in dogs and cats. A total of 23 patients with 24 lip avulsion injuries were included in the study. They were comprised of 11 dogs and 12 cats. The patients were generally young, with 68.2% under 3 years of age and 36.4% under 1 year of age. The most common known causes were animal bites (26.1%) and vehicular trauma (21.7%). In cats, the most common cause was vehicular trauma (25%). In dogs, the most common cause was an animal bite (45.4%). Bilateral rostral upper lip avulsion was most common in dogs (36.3%), whereas bilateral rostral lower lip avulsion was most common in cats (53.8%). Concurrent injuries were frequent in both species with tooth fractures reported in 34.7%. All lip avulsion injuries were treated via wound debridement and lavage followed by appositional repair with absorbable suture material. The most common short-term complication was wound dehiscence (21.4%). Surgical therapy was highly successful with no significant long-term complications reported. The results suggest that lip avulsion injuries are primarily seen in younger dogs and cats, usually result from vehicular trauma or animal bites, and are successfully managed with surgical repair.

## Introduction

Soft tissue injury of the maxillofacial region is common in dogs and cats and typically a result of trauma. Initial stabilization of the cardiovascular, respiratory and nervous systems are priority followed by general anesthesia for diagnostic exploration and surgical repair (1). Contaminated and infected wounds may render the animal unable or unwilling to eat. Depending on the extent and location of wounds, nasoesophageal, esophagostomy or gastrostomy tubes may be indicated to temporary bypass the oral cavity and allow it to heal. Lip avulsion injuries have been documented in humans, dogs, cats, and horses (2–8). Vehicular trauma and animal bites are frequently reported causes. The upper lip appears to be more involved in dogs after bite trauma whereas the lower lip seems to be more likely affected in cats that have been stepped on by a person (9).

The upper and lower lips form the rostral and most lateral boundaries of the oral vestibule. The upper lips join rostrally at the philtrum, and the upper and lower lips on either side join caudally at the commissure (10). There are small median frenula in the front of the labial mucosa of the upper and lower lips and unnamed strong lateral frenula distal to the canine teeth at the lower lips. The labial mucosa of the lips is continuous with the buccal mucosa of the cheeks; both continue as alveolar mucosa before meeting the gingiva at the mucogingival junction. A rich vascular network that includes the superior and inferior labial arteries, the infraorbital artery and mental arteries supplies the skin, mucosa, lips and cheeks, and bones and teeth. This rich vascular supply aids in healing as well as success of flap techniques. The inner layer of the cheeks consists mainly of the buccinator muscles, while the outer layer is formed by the orbicularis oris muscles. The deep muscles of the lips include the zygomaticus, superior and inferior incisivus, levator labii superioris, caninus, mentalis, and levator nasolabialis. All are innervated by the facial nerve. The sublingual tissues include the sublingual caruncles, lingual frenulum, sublingual folds, and the genioglossus and geniohyoideus muscles (11).

Lower lip avulsions usually are a result of caudally or laterally directed forces on the lower lip and chin, whereas upper lip avulsions are a result of dorsal or caudodorsal forces on the upper lip and nose. Avulsions tend to separate at the mucogingival junction or gingiva. Variations in severity can be seen based on the amount of force and the level at which the force acted upon. A significant pull on the mucosa and subcutaneous tissues of the lower lips from animal bites could result in major bilateral avulsion to the level of the lip commissures, whereas falling from a minimal height may only result in a minor rostral avulsion at the level of the mucogingival junction. Similarly, significant force on the upper lip can result in avulsion of the nasal cartilages and acute oronasal fistula formation.

Few reports of lip avulsion injuries exist in the veterinary literature. The purpose of this retrospective case series was to identify and describe the clinical presentation, signalment, causes, lesion location, treatment, and outcome of lip avulsion injuries in client-owned dogs and cats.

## Materials and methods

Medical records of the Matthew J. Ryan Veterinary Hospital of the University of Pennsylvania (MJR-VHUP) were reviewed from client-owned dogs and cats with reported lip avulsion injuries from 2001 to 2017. Searches were performed using an electronic database as well as case logs of the Dentistry and Oral Surgery Service.

Patients were eligible for inclusion regardless of treatment modalities and outcome. Cases were excluded if the lip avulsion occurred as a direct result of a maxillofacial fracture resulting in vertical tears of the gingiva and/or alveolar, labial or buccal mucosa. Cases with incomplete or stable bone fractures or mandibular symphyseal separation in the location of the lip avulsion were eligible for inclusion if there was a horizontal tear of the gingiva and/or alveolar, labial or buccal mucosa. Cases were excluded if the injuries were lip and/or cheek punctures or lacerations, rather than degloving wounds. Cases that suffered concurrent maxillofacial trauma in an area other than the location of lip avulsion were also eligible for inclusion.

Breed, gender, weight, age at time of presentation, potential cause of the lip avulsion, lesion location and extent, results of diagnostic tests when appropriate, treatments, and outcomes were recorded. Dental radiographs and clinical photographs were reviewed when available. Attempts were made to call the owners of all patients for follow-up information.

**Figure 1:**
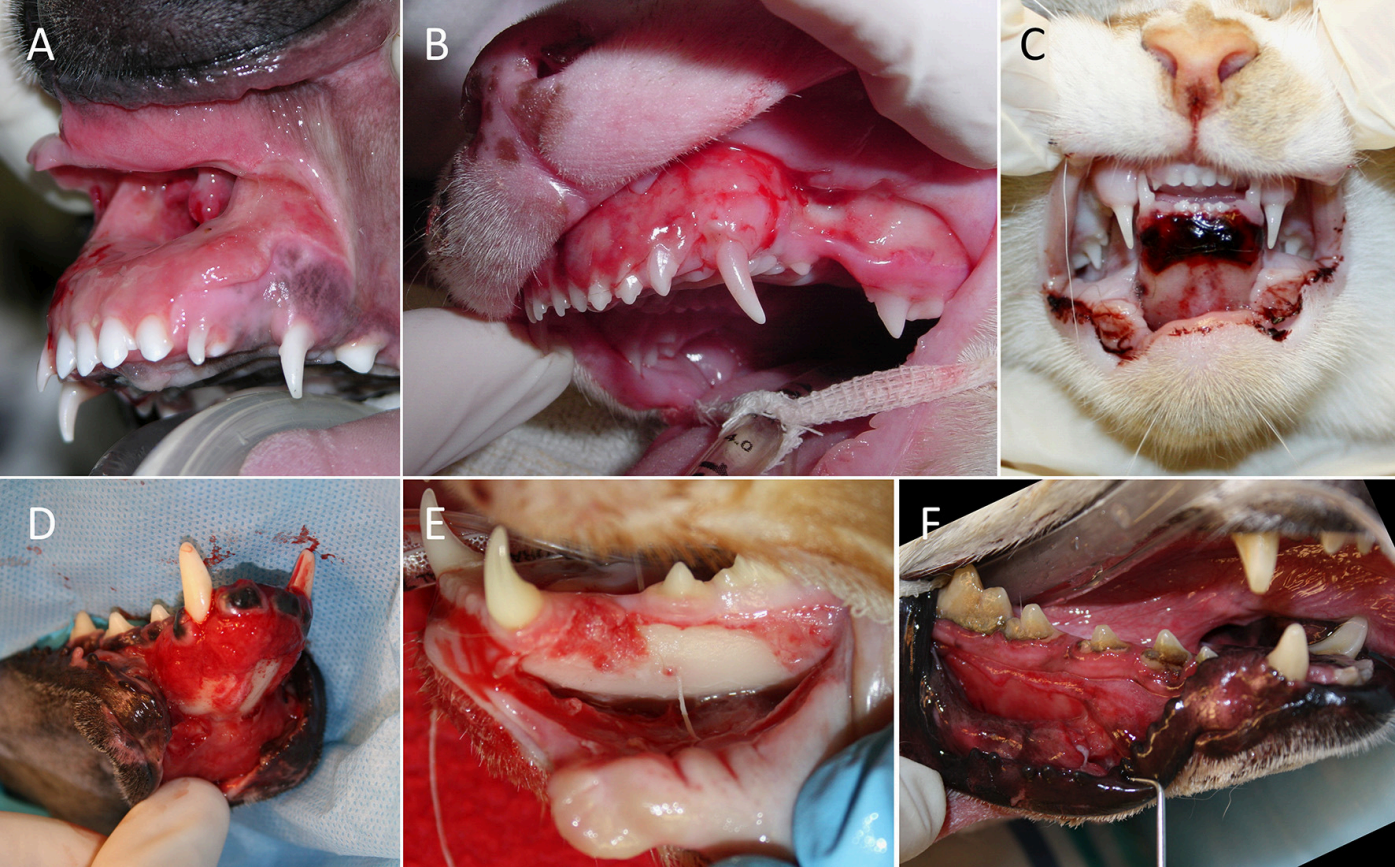
Categorization of lesion location in 23 dogs and cats with 24 lip avulsion injuries. **(A)** Bilateral rostral upper lip, **(B)** bilateral rostral upper lip with unilateral caudal involvement, **(C)** bilateral rostral lower lip, **(D)** bilateral rostral and caudal lower lip, **(E)** unilateral rostral and caudal lower lip, and **(F)** partial caudal lower lip avulsion.

Lip avulsion injury location was defined as upper or lower, unilateral or bilateral, and rostral or caudal (Figure 1). Bilateral lesions were those that crossed the midline, whereas unilateral lesions did not. Rostral lesions extended to the area of the canine tooth region, but not caudal to the lateral frenulum of the lower lip, and caudal lesions were limited to the premolar and molar tooth region. Caudal lesions were described as partial if the lesion was not continuous with a rostral lip avulsion.

## Results

**Figure 2:**
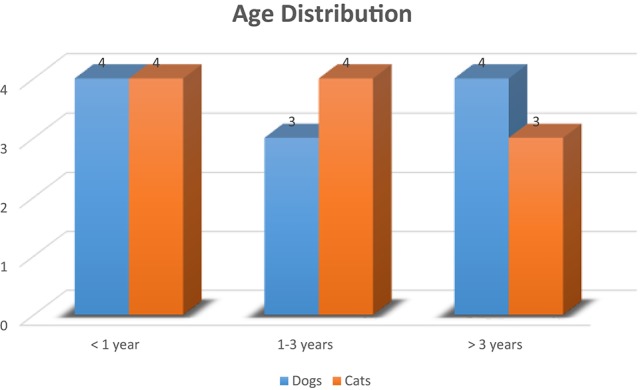
Age distribution of 22 dogs and cats with 23 lip avulsion injuries (age was not known for one patient).

A total of 23 patients with 24 lip avulsion injuries were included in the study. They were comprised of 11 dogs and 12 cats. All but one had a known age provided (Figure 2). The median age was 22 months (mean 34.8 months; lower quartile of 5 months, upper quartile of 63 months; range, 1–108 months). Patients were generally very young with the majority less than 3 years of age. Median weight for dogs was 8.3 kg (range, 1.9–16 kg) and for cats 4.1 kg (range, 0.6–6.6 kg). Dog breeds included American pit-bull terrier (2), Scottish terrier (1), boxer (1), miniature schnauzer (1), Dachshund (1), Australian shepherd (1), and mixed breed (4). All but one cat (a Persian) were domestic shorthair. Three dogs were male, of which two were intact. Eight dogs were female, of which one was intact. Nine cats were male, of which three were intact. Three cats were female, of which two were intact.

Causes for lip avulsion injuries included vehicular trauma, animal bites, falling from a height, being stepped on, and unknown trauma. Eight (34.8%) animals were stray or lived indoor/outdoor and were found with injuries. Of the known causes, animal bites were most common (26.1%) followed by vehicular trauma (21.7%). In cats, the most common cause was vehicular trauma (25%). In dogs, the most common cause was an animal bite (45.4%), with 80% resulting from conflicts with other dogs and one due to fighting with a raccoon. Concurrent injuries were found in some patients for each category of lip avulsion injury (Table 1).

**Table 1:** Causes of lip avulsion injury and concurrent trauma in dogs and cats.

	**Animal bite**	**Vehicular trauma**	**Being stepped on**	**Fallen from a height**	**Unknown**	**Other injuries present**
Dogs	45.4% (5/11)	18.2% (2/11)	0% (0/11)	18.2% (2/11)	18.2% (2/11)	81.8% (9/11)
Cats	8.3% (1/12)	25% (3/12)	16.7% (2/12)	0% (0/12)	50% (6/12)	83.3% (10/12)
Total	26.1% (6/23)	21.7% (5/23)	8.7% (2/23)	8.7% (2/23)	34.8% (8/23)	82.6% (19/23)

Location and severity of the lip avulsion injuries were variable, with the rostral upper lip bilaterally (25%) and the rostral lower lip bilaterally (29.3%) most commonly involved. Bilateral rostral/caudal lower lip lesions, caudal (partial) upper lip lesions, and caudal (partial) lower lip lesions were least common (Table 2). Bilateral rostral and unilateral caudal avulsions of the lower lip and unilateral rostral and caudal avulsions of the upper lip were not reported in the studied population. One cat had sustained both a rostral upper lip avulsion and a rostral lower lip avulsion. Bilateral rostral upper lip avulsion was most common in dogs (36.3%), whereas bilateral rostral lower lip avulsion was most common in cats (53.8%).

**Table 2:** Location of lip avulsion injuries in dogs and cats.

	**Bilateral rostral upper lip**	**Bilateral rostral lower lip**	**Bilateral rostral and caudal lower lip**	**Bilateral rostral upper lip with unilateral caudal involvement**	**Unilateral rostral and caudal lower lip**	**Partial caudal upper lip**	**Partial caudal lower lip**
Dogs	36.3% (4/11)	0% (0/11)	9.1% (1/11)	27.3% (3/11)	0% (0/11)	9.1% (1/11)	18.2% (2/11)
Cats	15.4% (2/13)	53.8% (7/13)	7.7% (1/13)	0% (0/13)	15.4% (2/13)	7.7% (1/13)	0% (0/13)
Total	25% (6/24)	29.3% (7/24)	8.3% (2/24)	12.5% (3/24)	8.3% (2/24)	8.3% (2/24)	8.3% (2/24)

Nineteen patients (82.6%) had injuries in addition to a lip avulsion. These included tooth fractures (34.8%), facial wounds and abrasions (34.8%), traumatic brain or nerve injury (21.7%), pulmonary contusion (13%), ocular injury (13%), other bony fractures (13%), incisive bone fracture (8.7%), mandibular symphyseal separation (8.7%), tongue laceration (8.7%), and traumatic palatal defect (4.3%). One patient had severe nasal tissue avulsion as a result of a dog bite. All patients with tooth fractures had one or more complicated crown fractures. The deciduous or permanent canine teeth composed 76.9% of the complicated crown fractures. All patients with mandibular symphyseal separation had experienced vehicular trauma. Additionally, all patients with tooth fractures and traumatic brain injury either had experienced vehicular trauma or trauma of unknown origin. Of the patients that had concurrent fractures of the upper or lower jaw, 75% (3/4) had experienced vehicular trauma, and 25% (1/4) had sustained facial bite wounds.

**Figure 3:**
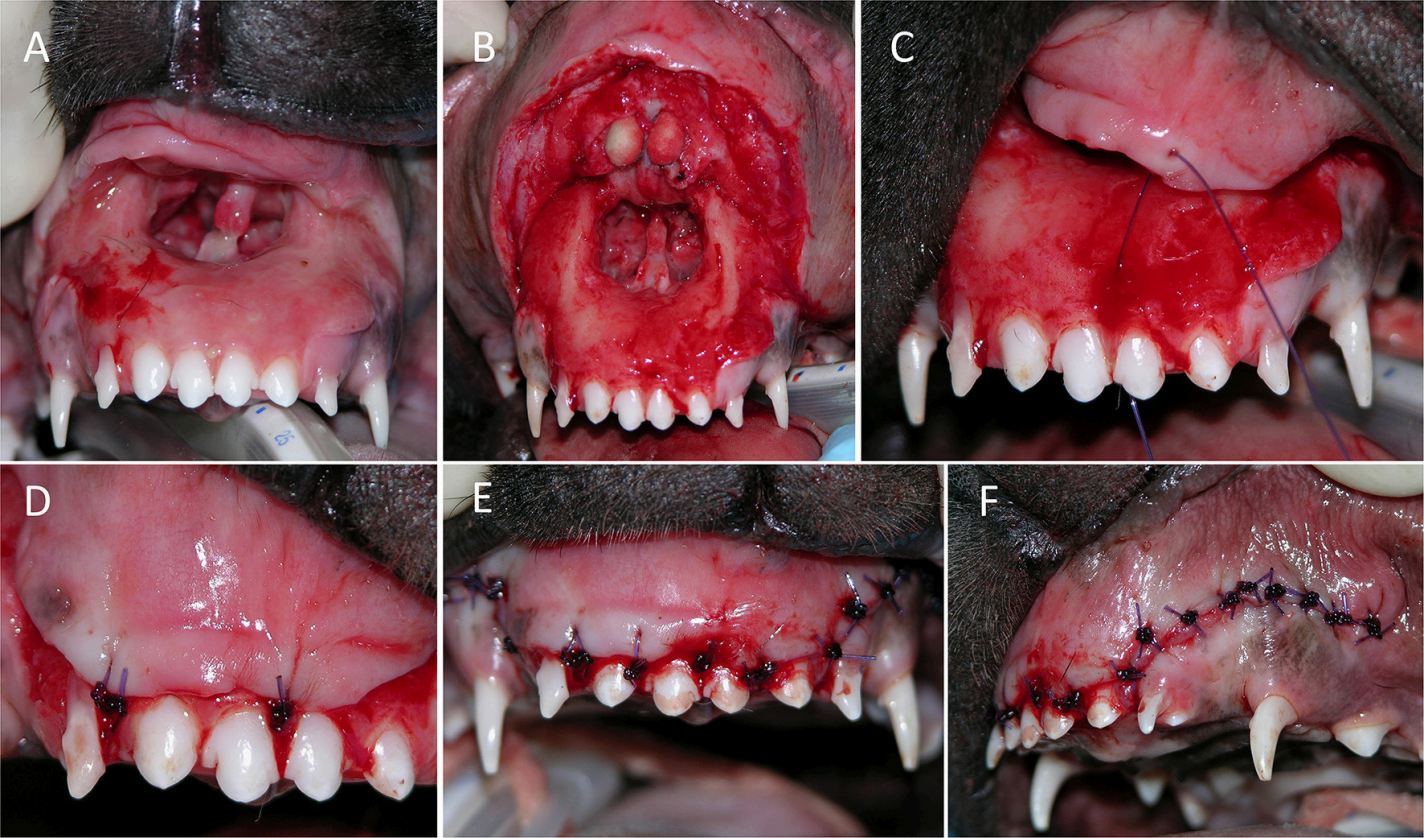
Treatment of a bilateral rostral upper lip avulsion in a dog. **(A)** Prior to debridement of oral tissues. **(B)** After debridement of oral tissues. **(C,D)** Simple interrupted sutures placed interproximally. **(E,F)** Front and side views showing complete wound closure.

**Figure 4:**
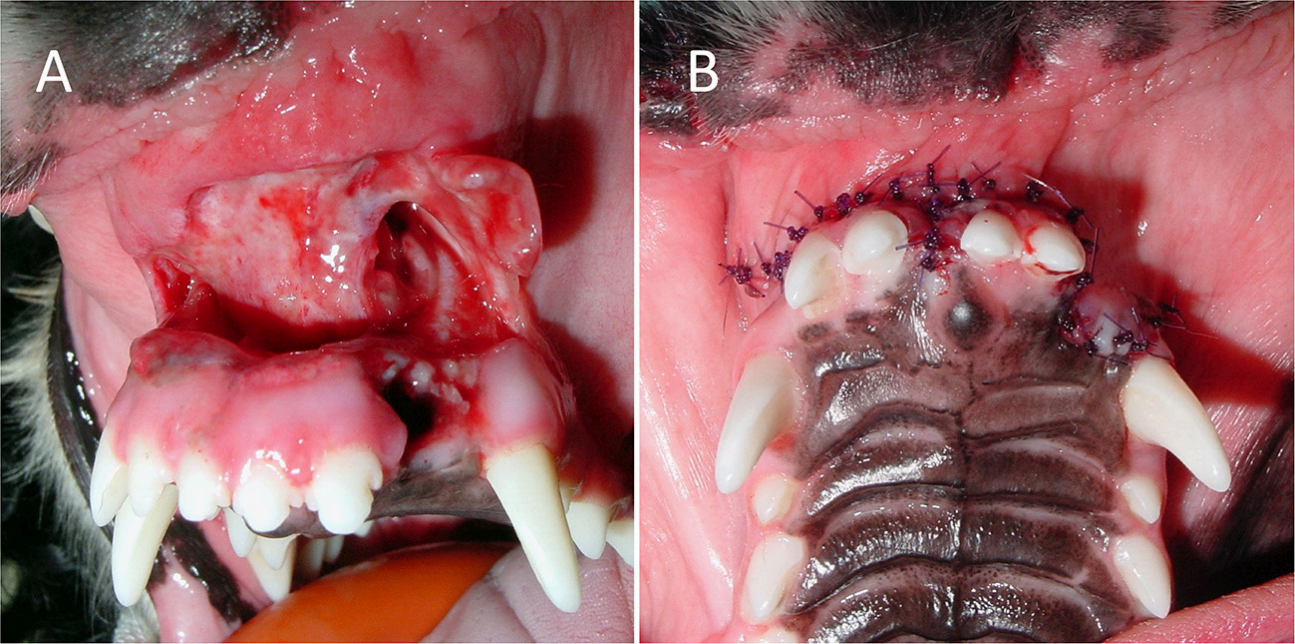
Treatment of a bilateral rostral upper lip avulsion in a dog. **(A)** After debridement and tooth extractions, **(B)** the wound was sutured closed in a simple interrupted pattern.

**Figure 5:**
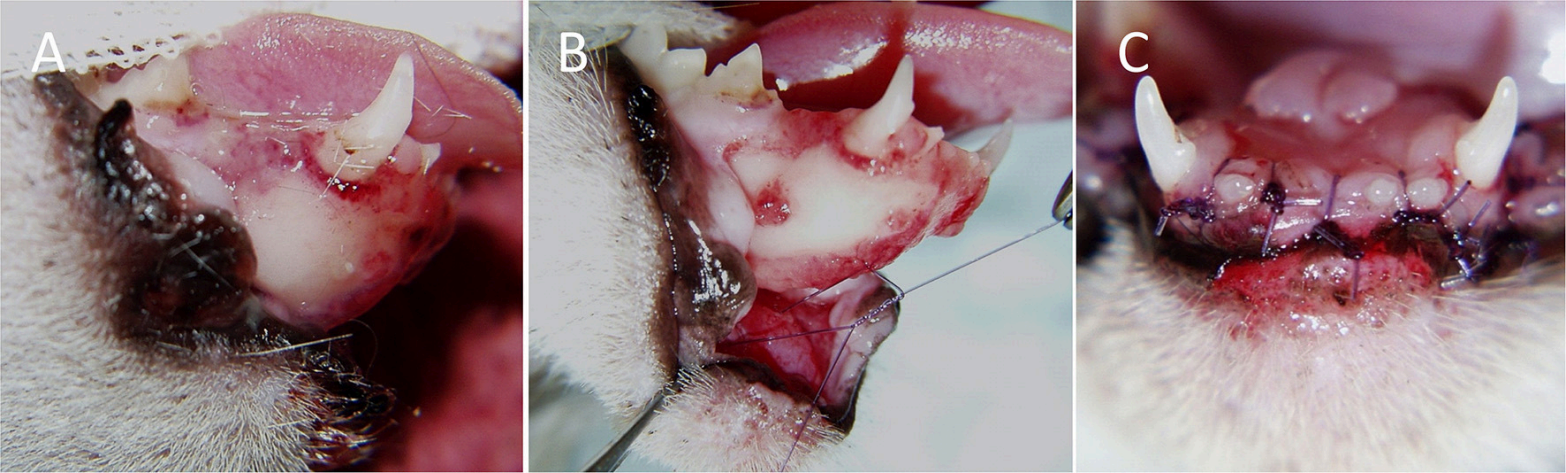
Treatment of a bilateral rostral lower lip avulsion in a cat. **(A)** Prior to debridement of oral tissues. **(B)** After debridement of oral tissues a tacking suture was placed to reduce dead space by anchoring the connective tissue of the lower lip to symphyseal connective tissue. **(C)** Final closure was accomplished using simple interrupted sutures placed interproximally.

**Figure 6:**
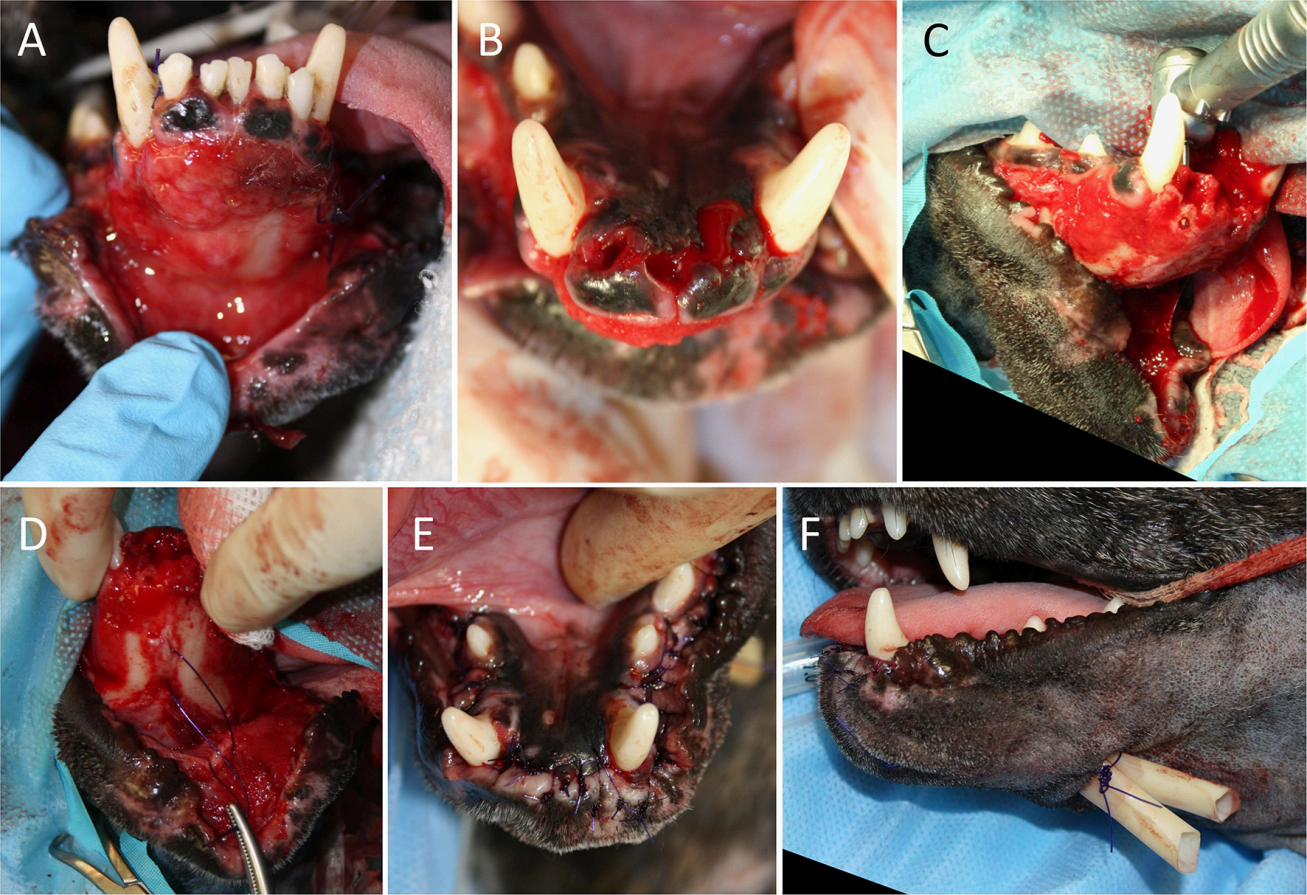
Treatment of a bilateral rostral and caudal lower lip avulsion in a dog. **(A)** Prior to debridement of oral tissues. **(B)** Following debridement of oral tissues and extraction of remaining mandibular incisor teeth. **(C)** Placement of an intraosseous guide hole. **(D)** Reduction of dead space by anchoring the connective tissue of the lower lip to the mandibular symphyseal connective tissue. **(E)** Final closure with simple interrupted sutures. **(F)** Penrose drain placed.

The mean time from known trauma or acquisition to surgery was 1.9 days (range, 0–7 days). All lip avulsion injuries were treated via wound debridement and lavage followed by appositional repair with simple interrupted and/or horizontal mattress sutures (occasionally around teeth for additional anchorage) using absorbable suture material (Figures 3, 4). Dead space was reduced using tacking sutures from subcutaneous tissue to intermandibular musculature or mandibular symphyseal connective tissue (Figure 5). Intraosseous sutures were used in two cases. Both of them had teeth extracted in the area of the lip avulsion injury to allow for better apposition of tissues (Figure 6). One of the cases treated in this manner already had three failed repairs of a bilateral rostral and caudal lower lip avulsion. Penrose drains were placed in two cases. Three cases had insufficient information in the medical records and dental charts to accurately describe the surgical technique used.

Analgesia was administered to all patients intraoperatively in the form of intravenous opioids, non-steroidal anti-inflammatory drugs, and/or regional nerve blocks using bupivacaine (16/23) or a combination of bupivacaine and lidocaine (2/23). Two patients did not receive any intraoperative nerve blocks. This information was unavailable in the remaining three patients. All but three patients received pain medications postoperatively and included oral tramadol, transmucosal buprenorphine, carprofen, deracoxib, gabapentin, and transdermal fentanyl patches.

Antimicrobial therapy was used in all patients. However, two patients only received perioperative intravenous antimicrobial therapy. Of these two patients, one was lost to follow-up, and the other had slight dehiscence noted that healed by second intention. Antimicrobials prescribed included oral amoxicillin-clavulanic acid (80.1%), clindamycin (14.3%), enrofloxacin (4.8%) and erythromycin (4.8%) as well as topical chlorhexidine gel (28.6%). One patient was prescribed amoxicillin-clavulanic acid and clindamycin simultaneously. Chlorhexidine gel, when used, was an adjuvant therapy used with a systemic antimicrobial. Therapeutic duration ranged from 5 to 21 days with a mean of 10.9 days.

**Figure 7:**
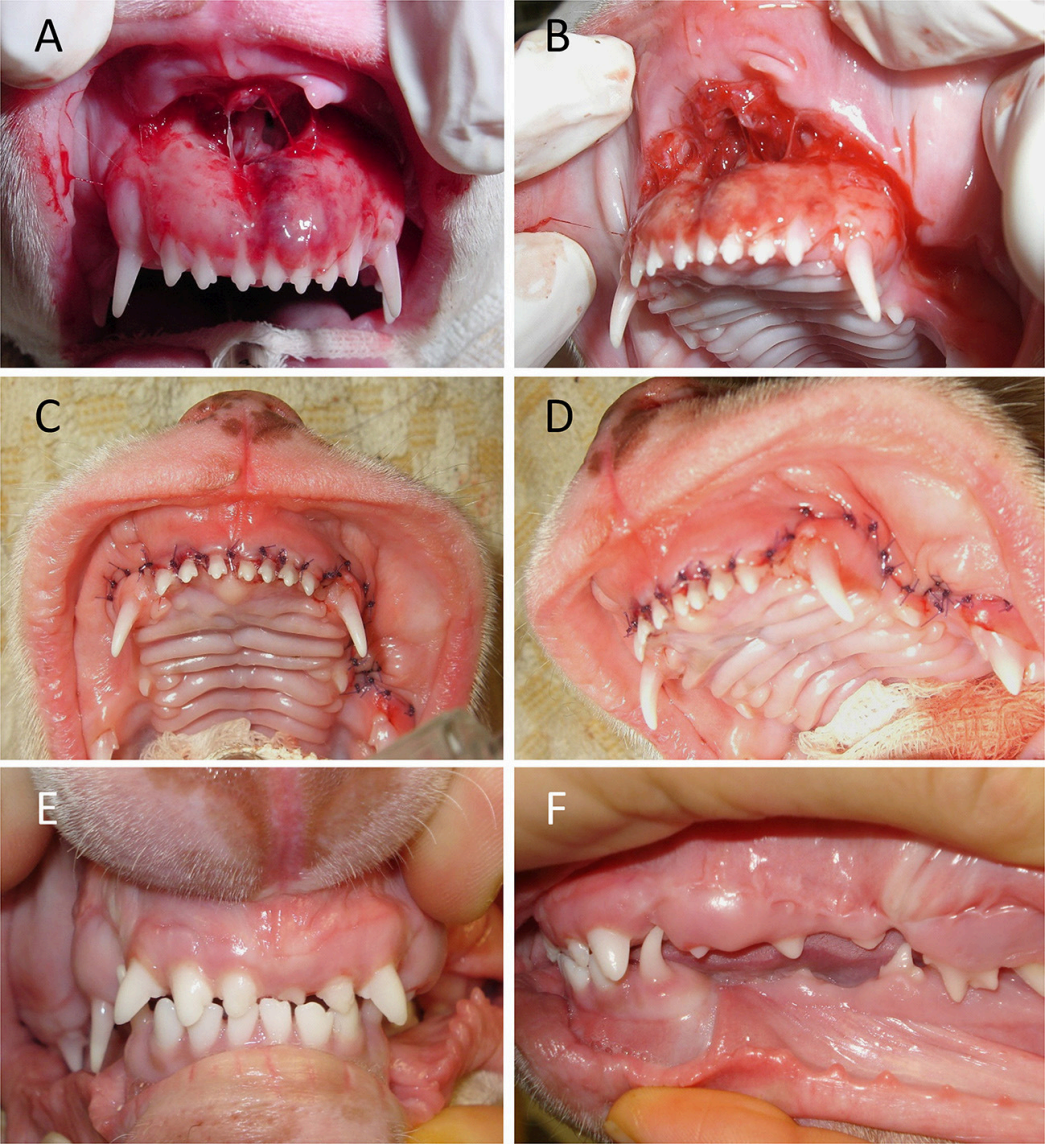
Outcome of bilateral upper lip avulsion injury with unilateral caudal involvement in an immature dog with deciduous dentition. **(A,B)** Prior to debridement of oral tissues. **(C,D)** Wound closure following debridement, rinsing, and suturing. **(E,F)** Healing of involved soft tissues confirmed 3 months postoperatively.

Of the 23 patients evaluated, 14 were available for short-term follow-up (mean 21.7 days; range, 2–57 days). The most common complication noted was wound dehiscence (*n* = 3; 21.4%), followed by postoperative infection (*n* = 1), tooth discoloration (*n* = 1), neurapraxia (*n* = 1), and dyspnea (*n* = 1). One dog had only a minor wound dehiscence that healed via second intention. The other two cases with wound dehiscence required surgical revision. They were cats with bilateral rostral lower lip avulsion as a result of vehicular trauma. One had a fracture through the alveolus in the area of the avulsion and subsequent extractions of teeth, and the other had a mandibular symphyseal separation. One dog had surgical repair at another location three times prior to presentation and was referred to our hospital after the third wound dehiscence; this case was not considered in the wound dehiscence data. Postoperative infection was seen in one cat 4 months after surgery to repair a bilateral lower lip avulsion; the cause of the initial trauma was unknown. Discoloration of the crown was seen in two deciduous incisor teeth of a dog that fell from a table, which resulted in a bilateral rostral and caudal upper lip avulsion injury. One young cat had no clinical signs other than a bilateral lower lip avulsion injury apparent on initial examination but was euthanized 2 days after surgery due to dyspnea and presumed laryngeal edema. All but the aforementioned feline patient survived the trauma and surgical repair, with 64.3% (9/14) experiencing a full recovery without complications (Figure 7). All patients received postoperative pain medication. Two cats that sustained severe orofacial trauma required temporary esophagostomy tube placement for nutritional support.

Of the 23 patients evaluated, eight were available for long-term follow-up data analysis (mean 66.5 months; range, 4.4–111 months). Owners did not report any issues with breathing, masticatory function, or oral discomfort/pain. No subsequent oral surgeries related to the original injuries were required. Two cat owners reported a change in cosmetic appearance with the affected lower lip drooping more toward the side of the initial trauma. Both of these cats had sustained unknown trauma to the rostral lower lip.

## Discussion

Lip avulsion injuries are sequelae of orofacial trauma and most commonly result from vehicular trauma or animal bites. Younger animals may be overrepresented due to their inquisitive and clumsy nature. Prognosis with surgical repair is excellent. Wound debridement and lavage followed by primary closure is the preferential treatment for lip avulsion injury. Closure via second intention is not recommended due to contracture of the tissues, long-term bone exposure, and potential loss of the oral vestibule (1). Special attention should be paid to tissues suspicious of undergoing necrosis that are very dark, gray, white, or those that are separating from underlying tissues. Debridement may be used in a staged approach to allow tissues to fully declare themselves before definitive therapy. Copious lavage is performed using sterile isotonic saline, a balanced electrolyte solution or dilute chlorhexidine (12). Lactated Ringer's solution is preferred by the authors due to potential toxicity to the canine fibroblasts seen *in vitro* with saline and 0.12% chlorhexidine solution (13, 14). Placement of a Penrose drain should be considered when dead space is difficult to reduce.

Early decontamination and administration of antibiotics have been recommended (12). The oral cavity's excellent blood supply and the antibacterial properties of saliva make infections after oral surgery rare. However, due to the highly contaminated nature of lip avulsion injuries antibiotic therapy is usually warranted. Postoperative antimicrobial therapy was used in 91.3% of cases (21/23). The most commonly used systemic antimicrobials included amoxicillin-clavulanic acid and clindamycin. Choice of antimicrobial and duration of use was clinician-dependent. However, selection was based on broad-spectrum coverage of the antimicrobial, degree of contamination, and severity of the wound. In one study, subgingival anaerobic bacteria in dogs with periodontal disease were most susceptible to amoxicillin-clavulanic acid, doxycycline and erythromycin (15). Aerobic bacteria seemed to be the most susceptible to amoxicillin-clavulanic acid, erythromycin, gentamicin, and sulfa-trimethoprim (15). Chlorhexidine is a cationic bisguanide, which allows it to bind to negatively charged surfaces in the mouth, such as the teeth and oral mucosa (16). It has broad-spectrum activity and is highly effective against Gram-positive organisms. A commercially available 0.12% gel formulation was used for the patients in this case series. The two patients that experienced wound dehiscence and required surgical intervention were both prescribed appropriate courses of amoxicillin-clavulanic acid. Oral cultures and sensitivity testing were not performed for any of our patients. Infection as a complication was documented in a single case 4 months after initially successful lip avulsion repair. The cat showed an abscess with deep pocketing in the area of the left lower lip frenulum. It was hypothesized that debris could have accumulated in this pocket because of atypical lip conformation after the initial surgery. Since this cat had access to the outdoors, an unrelated additional trauma could not be ruled out.

Several suture techniques exist for the repair of lip avulsion injuries, some incorporating the crowns of teeth or the bone in the area. The more extensive the lip avulsion injury, the more likely the repair will require additional surgical reinforcement to be successful. If teeth are adjacent, simple interrupted or horizontal mattress sutures can be placed around their crowns for anchorage. Knots should preferably be tied on gingiva or the lingual or palatal aspects of teeth (and away from the more delicate labial or buccal soft tissues). Alternatively, if teeth are missing, guide holes can be drilled through bone at the alveolar margin to pass suture material through. Upper lip avulsion injuries may present a challenge due to the relatively thin and tightly adhered gingiva, which also predisposes this area to tear (3). This may be more of concern in cats that lack the elasticity of lips and cheeks seen in dogs (10). The use of anchoring sutures around teeth of the upper jaw helps to reduce difficulties in surgical repair.

For lower lip avulsion injuries several tacking sutures can be placed, connecting the subcutaneous tissue of the torn lip with mylohyoid muscle in the intermandibular space and the submucosal tissue with the fibrocartilagineous tissue of the mandibular symphysis to reduce dead space and establish proper alignment. Suture material or wire could also be placed through ventral skin and mucocutaneous junction, looped around mandibular teeth, and then be brought back through the opposite side's mucocutaneous junction and ventral skin (12). This technique is similar to a cerclage wire for a mandibular symphyseal separation. Intravenous fluid tubing, a red rubber catheter or button can be used as a tension-relieving mechanism along the skin (4, 12). The suture material should be left relatively loose with this technique to avoid compromising the blood supply to the flap.

More extensive lower lip avulsion injuries may warrant the use of intraosseous placement of suture material or wire. Holes are drilled from labial/buccal to lingual through the mandible. Suture material or wire is inserted through these holes to loop around the crowns of teeth and connect externally along the skin at a level that approximates the normal lip conformation and tightened just enough to appose the soft tissues against the mandible (12). Care must be taken to avoid drilling into tooth roots (or permanent tooth buds in immature animals) when using this technique. A simple interrupted suture pattern is then used to appose the labial/buccal mucosa and gingiva (12). Closure of avulsed muscle and submucosal tissue will not only reduce dead space but also tension on the mucosal suture line. No attempts were made to realign nasal cartilages when they were involved in an upper lip avulsion injury. This procedure is technically challenging due to limited visibility to the surgical site during tissue maneuvering and the potential for malalignment and subsequent respiratory difficulties.

Absorbable monofilament suture material was used in all patients of this case series. The authors' preference is 4-0 or 5-0 poliglecaprone 25 with a swaged-on tapered round or reverse cutting needle. This suture material is preferred due to its excellent tensile strength, handling characteristics, absent capillarity and minimal tissue response (17). It has reliable and rapid degradation via hydrolysis, even in infected tissue, such that the tensile strength is decreased by 50% after 7 days and completely lost at 21 days. It is absorbed between 91 and 119 days (18, 19). Polydioxanone (PDS) can be used in the oral cavity when prolonged healing times are anticipated and extended tensile strength is needed (19). A swaged-on needle is preferred as it is less traumatic to tissues and easier to work with (18). Cutting needles were created for more dense tissues, but the sharp edge on the inside of their curve can cause tearing of tissues. Reverse cutting needles have a flat inner curve, but a third cutting edge on the outer surface which is directed away from the wound edge (17). Multifilament suture materials have greater tissue drag, capillarity and serve as a nidus for bacteria, and they are not recommended for use in oral surgery. Suture material should be chosen based on the type of procedure performed and desired length of time the suture needs to be functional (17). In addition, while no suture material to date satisfies all criteria, the material chosen should be non-allergenic, cause minimal tissue reaction, have good handling characteristics, knot security, and tensile strength (17, 19). Due to the impracticality of routine sedation or anesthesia that would be necessary to remove oral sutures in most veterinary patients, non-absorbable suture materials are not ideal for use in oral procedures. Because the same suture material was used in all patients, an association between incidence of dehiscence and suture material cannot be made in this case series. Dehiscence was likely a result of other factors including severity and location of injury, tissue handling, early decontamination, and concurrent injuries in the area of the lip avulsion injury.

Flap techniques should be considered for cases where a substantial amount of tissue has been lost at the time of the initial trauma or becomes necrotic post trauma. Rotation flaps, single-pedicle advancement flaps, and labial advancement flaps are examples. Restoration of function and maintaining normal occlusion are paramount goals in veterinary patients, with excellent cosmesis playing a smaller role. This is in contrast to human medicine where the cosmetic outcome is of high importance. Children and young adults commonly present for being bitten by dogs, causing significant lip avulsion injuries and facial disfiguration. Disfigurement despite adequate function can result in psychologic stress leading to loss of self-esteem, reduced socialization, and even depression (5, 6).

Lesions in humans either are allowed to heal by second intention, repaired via primary closure, or reconstructed via a myriad of flap and grafting techniques. If sufficient tissue and viable blood vessels are available, microvascular surgery to repair torn neurovascular bundles is the preferred therapy (6, 20, 21). This option gives the patient the highest chance at regaining function and sensation (17). Venous congestion and subsequent flap necrosis is a commonly reported sequelae of this technique; however, the use of leeches is often employed successfully for venous drainage (17–19). Healing by second intention has been advocated as the therapy of choice for younger patients with surgical revision of contracted scar tissue as necessary (5). The success of flap techniques have been described as more unpredictable and are generally reserved for cases with large defects, failed initial management, or lack of viable blood vessels (6).

The most common short-term complication seen in the study presented here was wound dehiscence. Most lip avulsions heal uneventfully if the tissues are properly handled, debrided and lavaged prior to wound closure. Otherwise, infection, abscess formation, necrosis and dehiscence may be seen. Failure to achieve tension-free closure will undoubtedly result in partial or full dehiscence, especially in the lower lip area where gravity and the bilateral lateral labial frenula create a more ventral pull on the tissues. In one dog with bilateral rostral and caudal lower lip avulsions, three unsuccessful attempts were made to repair the lesions. In that case, definitive surgery required removal of the mandibular incisors to allow for the creation of guide holes through the bone for placement of intraosseous sutures.

**Figure 8:**
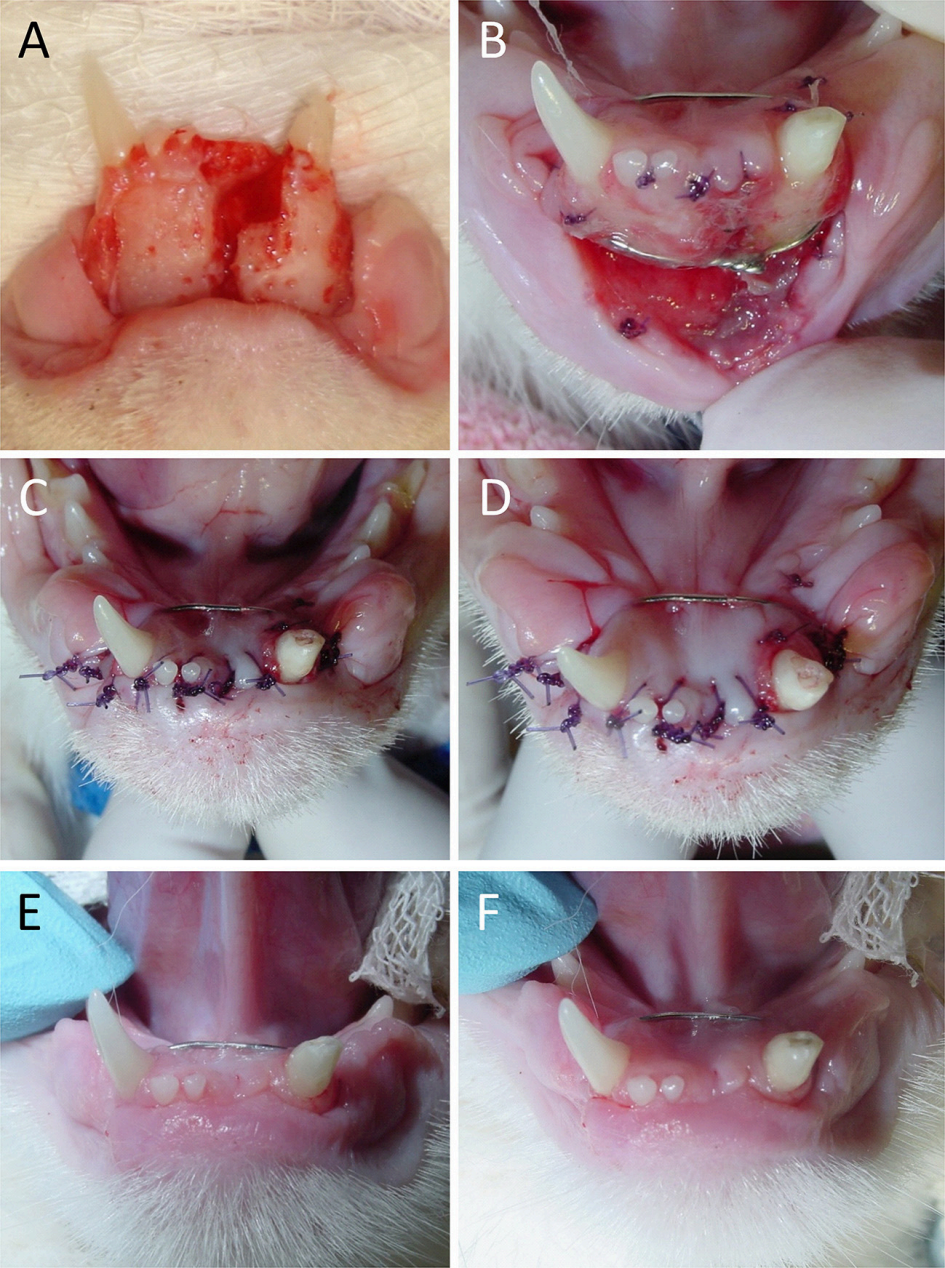
Outcome of bilateral lower lip avulsion injury in an adolescent cat with permanent dentition and mandibular symphysis separation. **(A)** After debridement of oral tissues at the time of presentation. **(B)** Dehiscence noted 6 days after the initial surgery. **(C,D)** Wound closure following rinsing and placement of additional tension-relieving suture. **(E,F)** Healing of involved soft tissues and the mandibular symphysis confirmed 1 month postoperatively.

Concurrent maxillofacial injuries are common and must be stabilized to aid in the success of lip avulsion repair (7, 12). Interestingly, all three of the cases with wound dehiscence had either a bone fracture in the area or mandibular symphyseal separation. It is possible, but unlikely, that unaddressed instability was a contributing factor. One of the cases had an incomplete mandibular fracture only affecting the alveolus of a tooth in the area. Adjacent teeth were extracted to allow for making guide holes through the bone and placing intraosseous sutures. This patient suffered from postoperative neurapraxia, defined as a temporary interruption of nerve conduction (22). This was suspected to affect branches of the trigeminal nerve based on electromyogram testing and could have caused additional tension on the surgery site of the dropped lower jaw. The other case with wound dehiscence and concurrent bone fracture had a hairline fracture of the incisive bone that did not require treatment as deemed by the attending clinician of that case. Mandibular symphyseal separations were always repaired via a circumferential wire cerclage. With regards to the wound dehiscence seen in a cat with bilateral rostral lower lip avulsion and concurrent mandibular symphyseal separation, the cerclage wire used to repair the symphyseal separation was placed with the twisted knot in a ventral location, possibly causing increased tension on the labial mucosa. Surgical repair was successful after additional tension-relieving sutures were placed (Figure 8). The twist could have been placed in a lateral position to reduce tension on the suture line. In the presence of concurrent bony instability, stabilization techniques such as interdental wiring and composite splint placement, cerclage wiring, interfragmentary wiring, miniplate placement, maxillomandibular fixation or supportive muzzling techniques should be considered. On the other hand, other patients in our study with significant concurrent injuries such as nasal avulsion and mandibular symphyseal separation went on to heal without incident, suggesting the incidence of wound dehiscence cannot be explained by bony instability alone. Cases with wound dehiscence had 1–2 days elapse from the time of trauma to surgery, and two out of three were treated with postoperative antibiotics. Other factors, such as poor owner compliance with feeding and activity restrictions, flap tension, improper debridement, tissue necrosis, infection or self-trauma cannot be ruled out.

In the present case series, all bilateral rostral lower lip avulsions were seen in cats. This corroborates with cats reported in the veterinary literature that were hit by a car, stepped on by the owner, or suffered unknown trauma (7, 8, 10, 14). Cats with outdoor access are more vulnerable to vehicular trauma. Due to their relatively smaller body size compared with dogs, they may experience more significant traumatic contact with both the vehicle and road substance resulting in more significant injuries. Degloving of the lower lip may result from drag on the lower jaw when sliding on the road. Lower lip avulsion injury can also be seen more commonly in kittens that were stepped on and then attempted to pull away. Bilateral rostral upper lip avulsions were more common in dogs experiencing bite trauma by another dog.

Several patients had concurrent dental injuries that included complicated crown fractures, complicated crown-root fractures, an uncomplicated crown fracture, and retained root fragments. Extraction was the most common treatment and was performed at the time of initial surgery in all patients except for one case, which had further treatment delayed due to excessive anesthesia time. Two tooth fractures in two patients were treated with vital pulp therapy. Concurrent orofacial injury was very common, warranting a thorough examination prior to surgical repair of the lip avulsion injury. Dental and head radiography can be helpful but often is lower yield for injuries of the maxillae, nasal bones, and temporomandibular joints (TMJs) due to summation of other structures. If concurrent bony trauma or TMJ involvement is suspected, computed tomography (CT) would be recommended.

The most common risk factor for development of lip avulsion injury in cats appeared to be an indoor/outdoor lifestyle. Of the six cats suffering unknown trauma, five were outdoor or stray cats. This makes them more liable to interact with other animals, especially territorial stray cats. They also are at higher risk of vehicular trauma and falls. Bite wounds, overwhelmingly from other dogs, were the most common cause of lip avulsion injury in dogs. Most of the bite wounds were from neighbor's dogs or those encountered on walks, rather than housemates. Risk factors for dogs included exposure to off-leash dogs, being off-leash themselves, or situations where multiple dogs were exposed to one another such as in dog parks and boarding facilities.

Postoperative treatment may include a soft diet for 10–14 days, restricting the patient from chew toys or hard treats, antibiotic therapy (amoxicillin/clavulanic acid or clindamycin), oral chlorhexidine (in rinse or gel form), analgesic medications (non-steroidal anti-inflammatory drugs, tramadol, gabapentin, opioids), and an Elizabethan collar if necessary. Tape muzzles can be considered if a lower lip avulsion injury was extensive to prevent excessive tension on the sutured wound. None of the patients in this series were treated with a tape muzzle. A 2-week recheck examination is advised to monitor the healing of oral soft tissues. An anesthetized oral examination with dental radiographs is recommended 6–12 months after the initial presentation and treatment to monitor endodontic and periodontal health of teeth previously traumatized (23).

Lip avulsion injuries are a common result of vehicular, stepped on, and bite wound trauma. Prognosis is excellent with early debridement, lavage and tension-free wound closure. Concurrent orofacial injury is very common, necessitating a thorough examination and the use of diagnostic modalities such as dental radiography and CT. The patients of the present case series were generally less than 3 years of age. Cats with rostral lower lip avulsions were overrepresented, whereas rostral upper lip avulsions were more common in dogs.

## Author contributions

KS: Acquisition of data, analysis and interpretation of data, drafting of manuscript, revision of manuscript. AR: Study concept and design, acquisition of data, critical revision of manuscript for important intellectual content.

### Conflict of interest statement

The authors declare that the research was conducted in the absence of any commercial or financial relationships that could be construed as a potential conflict of interest.
