# Corrigendum: Clinical Presentation, Causes, Treatment, and Outcome of Lip Avulsion Injuries in Dogs and Cats: 24 Cases (2001–2017)

**DOI:** 10.3389/fvets.2018.00183

**Published:** 2018-07-31

**Authors:** Kelly M. Saverino, Alexander M. Reiter

**Affiliations:** Department of Clinical Studies, Dentistry and Oral Surgery Service, Matthew J Ryan Veterinary Teaching Hospital, University of Pennsylvania, Philadelphia, PA, United States

**Keywords:** lip, avulsion, degloving, canine, dog, feline, cat, trauma

In the original article, there was an error. We accidentally stated that non-absorbable monofilament suture material was used in all patients of this case series. That opposite is actually true. A correction has been made to the Discussion, Paragraph 6:

Absorbable monofilament suture material was used in all patients of this case series. The authors' preference is 4-0 or 5-0 poliglecaprone 25 with a swaged-on tapered round or reverse cutting needle. This suture material is preferred due to its excellent tensile strength, handling characteristics, absent capillarity and minimal tissue response (17). It has reliable and rapid degradation via hydrolysis, even in infected tissue, such that the tensile strength is decreased by 50% after 7 days and completely lost at 21 days. It is absorbed between 91 and 119 days (18,19). Polydioxanone (PDS) can be used in the oral cavity when prolonged healing times are anticipated and extended tensile strength is needed (19). A swaged-on needle is preferred as it is less traumatic to tissues and easier to work with (18). Cutting needles were created for more dense tissues, but the sharp edge on the inside of their curve can cause tearing of tissues. Reverse cutting needles have a flat inner curve, but a third cutting edge on the outer surface which is directed away from the wound edge (17). Multifilament suture materials have greater tissue drag, capillarity and serve as a nidus for bacteria, and they are not recommended for use in oral surgery. Suture material should be chosen based on the type of procedure performed and desired length of time the suture needs to be functional (17). In addition, while no suture material to date satisfies all criteria, the material chosen should be non-allergenic, cause minimal tissue reaction, have good handling characteristics, knot security, and tensile strength (17,19). Due to the impracticality of routine sedation or anesthesia that would be necessary to remove oral sutures in most veterinary patients, non-absorbable suture materials are not ideal for use in oral procedures. Because the same suture material was used in all patients, an association between incidence of dehiscence and suture material cannot be made in this case series. Dehiscence was likely a result of other factors including severity and location of injury, tissue handling, early decontamination, and concurrent injuries in the area of the lip avulsion injury.

The authors apologize for this error and state that this does not change the scientific conclusions of the article in any way.

The original article has been updated.

## Conflict of interest statement

The authors declare that the research was conducted in the absence of any commercial or financial relationships that could be construed as a potential conflict of interest.

